# Bioactive Markers Based Pharmacokinetic Evaluation of Extracts of a Traditional Medicinal Plant, *Piper sarmentosum*

**DOI:** 10.1093/ecam/nep143

**Published:** 2011-06-23

**Authors:** Khalid Hussain, Zhari Ismail, Amirin Sadikun, Pazillah Ibrahim

**Affiliations:** Department of Pharmaceutical Chemistry, School of Pharmaceutical Sciences, Universiti Sains Malaysia, Pulau Pinang 11800, Malaysia

## Abstract

*In vitro* assays are economical and easy to perform but to establish relevance of their results to real clinical outcome in animals or human, pharmacokinetics is prerequisite. Despite various *in vitro* pharmacological activities of extracts of *Piper sarmentosum*, there is no report of pharmacokinetics. Therefore, the present study aimed to evaluate ethanol extract of fruit of the plant in dose of 500 mg kg^−1^ orally for pharmacokinetics. Sprague-Dawley rats were randomly divided into groups 1, 2, and 3 (each *n* = 6) to study absorption, distribution and excretion, respectively. High performance liquid chromatography (HPLC) with ultraviolet detection was applied to quantify pellitorine, sarmentine and sarmentosine in plasma, tissues, feces and urine to calculate pharmacokinetic parameters. Pellitorine exhibited maximum plasma concentration (*C*_max_) 34.77 ng mL^−1^ ± 1.040, time to achieve *C*_max_ (*T*_max_) 8 h, mean resident time (MRT) 26.00 ± 0.149 h and half life (*t*_1/2_) 18.64 ± 1.65 h. Sarmentine showed *C*_max_ 191.50 ± 12.69 ng mL^−1^, *T*_max_ 6 h, MRT 11.12 ± 0.44 h and *t*_1/2_ 10.30 ± 1.98 h. Sarmentosine exhibited zero oral bioavailability because it was neither detected in plasma nor in tissues, and in urine. Pellitorine was found to be distributed in intestinal wall, liver, lungs, kidney, and heart, whereas sarmentine was found only in intestinal wall and heart. The cumulative excretion of pellitorine, sarmentine and sarmentosine in feces in 72 h was 0.0773, 0.976, and 0.438 **μ**g, respectively. This study shows that pellitorine and sarmentine have good oral bioavailability while sarmentosine is not absorbed from the gastrointestinal tract.

## 1. Introduction

Pharmacokinetics, the action of body on the drug, includes absorption, distribution, metabolism and excretion. Therapeutic outcome depends on the rate and extent at which drug reaches at the site of action, bioavailability. Pharmacokinetic parameters help to establish bioequivalence in-between formulations and to understand toxicology, drug exposure. Pharmacokinetic studies of herbs may also assist physicians in prescribing drugs safely and effectively to those patients who are consuming herbal products, because herbs may synergies or antagonise the drugs, herb-drug interactions [1, 2].

Since long, natural product scientists have been studying pharmacodynamics, the action of herbs on the body but less attention has been paid to study the effect of body on herbs. This has been witnessed by a study indicating only few pharmacokinetic reports on herbal preparations [3]. Unlike pharmaceuticals, pharmacokinetics of herbal products, mixture of known and unknown components, is always challenging due to their complexity and unavailability or inadequacy of standards and methods. Moreover, lack of pharmacokinetic studies is a biggest hindrance in the modernization of herbal products because there is no way to establish bioequivalence between products prepared by modified method and the original method [4]. Different types of marker compounds, characteristics to a particular plant, can be used to study the pharmacokinetics of these preparations. Using marker compounds, few herbal products such as *Ginkgo biloba, Allium sativum, Ephedra sinica*, *Artemesia annua*, and so forth, have been investigated for pharmacokinetics [5–8].

Pharmacokinetic studies are of prime importance prior to clinical trials of herbal products to make these remedies evidence-based drugs. The importance of pharmacokinetics of herbal products has also been emphasized in the literature [9]. Keeping this in view, a commercially important medicinal plant, *Piper sarmentosum*, has been selected in this study to evaluate its extracts for pharmacokinetics.

*Piper sarmentosum* is a tropical plant, used traditionally in South-East Asian region to cure various ailments [10–13]. The plant has also been investigated extensively for a number of pharmacological activities such as anti-amoebic [14], antibacterial [15], anti-TB [16], anti-neoplastic [12], neuromuscular blocking [17], hypoglycemic [18], anti-malarial [19], antioxidant [16, 20, 21] and antiangiogenic [22]. Based on these activities, from extracts of the plant various products are being manufactured and are being sold as nutraceuticals nowadays. Despite these developments, there is no report about pharmacokinetic studies on these extracts. These studies are prerequisite to understand whether the extracts are absorbed from gastrointestinal tract or not. The plant is reported to have a number of biologically active amides [23–25] and amongst these, we have selected pellitorine, sarmentine and sarmentosine to develop and validate a high performance liquid chromatography (HPLC) method for their simultaneous quantification, and to apply the method to study pharmacokinetics of ethanol extracts of fruit of the plant.

Two approaches, non-compartment and compartment model, are commonly used to evaluate the pharmacokinetic profile of a compound. Compartment models such as one compartment model, two- and three-compartment model are associated with more assumptions as compared with non-compartment model. Therefore, we have used non-compartment model in this study to evaluate pharmacokinetic profile of the markers in the extract using Trapezoidal rule [26].

## 2. Methods

### 2.1. Animals and Grouping

Male Sprague-Dawley rats weighing 313 ± 17 g, taken from the Animal House of the Universiti Sains Malaysia, Pulau Penang, were housed in standard cages in animal transit room of the School of Pharmaceutical Sciences, for 7 days to acclimatise. Standard pellet diet (Gold Coin, Penang, Malaysia) was given and tap water was supplied *ad libitum*. Animals were divided into three groups (*n* = 6). Group 1 was used to study oral absorption while Group 2 was used to evaluate tissue distribution. Group 3 was further divided into two sub-groups (*n* = 3) namely subgroup E and subgroup C. The animals of subgroup-E were used to study excretion of markers in feces and urine while subgroup C served as control. The study protocol was approved by the Animal Ethical Committee of the Universiti Sains Malaysia; vide reference #USM/PPSF/50 (009) Jld.

### 2.2. Preparation Extract and Dose

The fruit of the plant collected from the Botanical Garden of the School of Pharmaceutical Sciences, Universiti Sains Malaysia, was authenticated by Prof. Dr Zhari Ismail, Herbal Secretariat, School of Pharmaceutical Sciences, Universiti Sains Malaysia, where a voucher specimen was deposited vide reference No. 0071/06. The fruit was cleaned, sliced into small pieces, dried at 40°C and pulverized. The pulverized fruit material (50 g) was extracted twice with 300 mL ethanol by reflux for 1 h. The extract was filtered and dried *in vacuo* at 40°C. The markers were quantified in the extract by HPLC before preparing the dose, which was prepared by suspending the extract in a mixture of water and polyethylene glycol (PEG) 400 in a ratio of 1 : 1 v/v to get final concentration 100 mg mL^−1^.

### 2.3. Collection of Blood Samples for Absorption Studies

A dose of 500 mg kg^−1^ was administered orally to six overnight fasting rats of Group 1. Blood samples (0.5 mL) were collected from tail vein [27, 28] in EDTA coated tubes (Becton Dickinson and Company) at 0 min (pre dose), 0.5, 1, 2, 4, 6, 8, 12, and 24 h. The tubes containing blood were centrifuged at 2500 rpm at 10°C for 10 min to get plasma, which was then stored at −80°C until analyzed.

### 2.4. Sampling for Tissue Distribution Studies

A dose of the extract (500 mg kg^−1^) was administered orally to six overnight fasting rats of group 2 and food was withheld for further 1 h. Blood samples (0.5 mL) were collected from the tail vein at 0 min (pre-dosing) and 6 h, then the animals were sacrificed to get tissues such as intestine, liver, lungs, kidney and heart. Blood samples were centrifuged at 2500 rpm at 10°C for 10 min to get plasma samples, which were then stored at −80°C until analyzed. Frozen tissues were used to prepare 5% homogenate in 0.15 M potassium chloride. These homogenates were centrifuged at 2500 rpm at 10°C for 10 min and the supernatant was stored at −80°C until analyzed.

### 2.5. Collection of Urine and Excreta

A dose of the extract (500 mg kg^−1^) was administered orally to overnight fasting rats of subgroup-E and food was withheld for further 1 h. The animals of subgroup-C received the equivalent amount of vehicle which was used to prepare the dose, and served as control. The animals were housed in metabolic cages to collect urine and feces. The samples were collected at 0 min (pre-dosing) and subsequently at 5, 10, 24, 48, and 72 h. The samples were then extracted according to the protocol mentioned below and the extracted samples were stored at −80°C until analyzed.

### 2.6. Extraction of the Markers from Plasma, Tissues, Urine and Feces

#### 2.6.1. Plasma

Rat plasma (500 *μ*L) taken in centrifuge tube was mixed with acetonitrile (100 *μ*L) by vortex for 5 s. Then 1 mL ethyl acetate was added and mixed by vortex for 5 s, afterwards the tube was centrifuged at 3000 rpm for 5 min at 10°C. The supernatant was collected and dried with stream of nitrogen, and the residue was reconstituted with 500 *μ*L mobile phase.

#### 2.6.2. Urine

One-milliliter urine was taken in centrifuge tube containing 1 mL ethyl acetate. The tube was vortex for 5 s, centrifuged at 3000 rpm at 10°C for 5 min and the non-aqueous layer was collected, dried with stream of nitrogen, and the residue was reconstituted with 500 *μ*L mobile phase.

#### 2.6.3. Tissues

Three milliliter of 5% liver homogenate prepared in 0.15 M potassium chloride was taken in a centrifuge tube containing 200 *μ*L acetonitrile. The tube was vortex for 5 s and after adding 2 mL ethyl acetate, tube was vortex again for 5 s. Then the tube was centrifuged at 3000 rpm at 10°C for 5 min, supernatant was collected and dried, and the residue was reconstituted with 500 *μ*L mobile phase.

### 2.7. Fecal Matter

Wet fecal matter (500 mg) was dissolved in 2 mL ethyl acetate, vortex for 5 s and centrifuged at 3000 rpm for 5 min. The supernatant was collected and dried, and the residue was reconstituted with 500 *μ*L mobile phase.

All the samples were filtered through 0.45 *μ*m polytetrafluoroethylene (PTFE) syringe filter (Whatman, Maidstone, England) and kept in HPLC vials.

### 2.8. Chromatography and Quantification of the Markers

Standards (pellitorine, sarmentine and sarmentosine) previously isolated from fruit of *P. sarmentosum* were used to prepare mix standard stock solution as: 300 *μ*g pellitorine, 300 *μ*g sarmentosine and 200 *μ*g of sarmentine were dissolved in 1 mL methanol. The stock solution was further diluted with mobile phase to get a series of mix working standard solutions containing pellitorine and sarmentosine 0.03–3.00 *μ*g mL^−1^ and sarmentine 0.02–2.00 *μ*g mL^−1^.

All the samples were analyzed using HPLC system (1100 series, Agilent Technologies, Waldronn, Germany) equipped with degasser (G1379 A), quaternary pump (G1311 A), auto sampler (G1313 A), column oven (G1316 A) and ultraviolet (UV) detector (G 1314 A).

The samples (15 *μ*L) were eluted by an isocratic mobile phase comprising of methanol : water : acetonitrile (80 : 15 : 5 v/v) at flow rate of 1 mL min^−1^ through column (Hiber Rt 250-4, LiChrosorb RP 18, 10 *μ*m, Agilent Technologies), which was maintained at 25°C. The elution time was 15 min and the detection was carried out at 260 nm by operating the detector in a sensitivity range of 0.005 AUFS with output of 15 mV. The data acquisition was performed by ChemStation version A. 08.03 and the markers were quantified by external standard method.

### 2.9. Determination of Pharmacokinetic Parameters

Analytical data of each rat was used to plot plasma concentration versus time. Total area under the plasma concentration versus time curve (AUC_0-∞_) was calculated using Trapezoidal rule [26] which is given as follows:

(1) $$\text{AU}{\text{C}}_{0-\infty }=\sum_{}({\text{AUC}}_{0-1}+{\text{AUC}}_{1-\text{last}}+{\text{AUC}}_{\text{last}-\infty }),$$

where AUC_last-∞_ = *C*_last_/*K*_el_.

A plot of product of concentration and time (CT) versus time was used to calculate area under first moment curve (AUMC). Mean resident time (MRT) was determined by dividing AUMC_0-∞_ with AUC_0-∞_ The maximum plasma concentration *C*_max_ (ng mL^−1^) and the time to achieve *C*_max_, *T*_max_ (h), were obtained directly from the data. The elimination rate constant *K*_el_ (h^−1^) was calculated by linear regression from the terminal phase of the plot of plasma concentration versus time using following equation:

(2) $$K_{\text{el}}=\frac{\ln\;C_{1}-\ln\;C_{2}}{T_{2}-T_{1}}.\;$$

The half-life *t*_1/2_ (h) was calculated by dividing 0.693 with *K*_el_. The clearance (Cl) and the volume of distribution (VD) were calculated from the equations given as follows:

(3) $$\text{Cl}=\frac{\text{Actual}\;\text{ dose}\;\text{ administered}}{{\text{AUC}}_{0-\infty }},$$

(4) $$\text{VD}=\frac{\text{Clearence (Cl)}}{\text{Elimination constant}(K_{\text{el}})}.$$

### 2.10. Statistical Analysis

Each sample has been analyzed in triplicate and the results are presented as mean ± standard deviation (SD). The values of pharmacokinetic parameters for absorption and distribution are the average of six rats ± SD while the excretion values are the average of three rats ± SD.

## 3. Results

### 3.1. Validation of HPLC Method of Analysis

The results shown in (Table 1) indicate the calibration data, limit of detection (LOD) and limit of quantification (LOQ) of pellitorine, sarmentine and sarmentosine. The method has been found linear over the whole range of samples investigated with correlation coefficients (*R* ^2^) ranging from 0.9997 to 1.0000 with SD <5%. It is obvious in the table that LOD values of pellitorine, sarmentine and sarmentosine are 3.00, 3.00, and 20.00 ng mL^−1^, respectively, while 10.00, 10.00 and 80.00 ng mL^−1^, respectively, have been taken as LOQ at signal to noise ratio 10 : 1. Extraction recovery values of pellitorine, sarmentine and sarmentosine are found to be 95.52–97.50, 96.23–98.43, and 96.47–100%, respectively, with relative SD <5%. Intra- and inter-day analysis accuracy values of the markers are 97.97–100.19% with relative SD <5%. These results have indicated that the method is reliable, repeatable and reproducible because the recovery of the markers is not compromised in intra- and inter-day analysis.

### 3.2. Content of the Markers in the Extract, Plasma, Tissues, Urine, and Feces

Before the preparation of dose, the content of markers, pellitroine, sarmentine and sarmentosine, were determined in the extract by HPLC and found to be 52.10, 13.10, and 0.21 mg g^−1^, respectively. This standardized extract was administered orally in a dose of 500 mg kg^−1^ to rats and the samples obtained at specified intervals were analyzed in triplicate by HPLC to quantify the markers in plasma, tissues, urine, and feces. These values were then used to calculate different pharmacokinetic parameters. The chromatograms of mix standard solution, the extract, blank plasma, markers in plasma and tissues, whereas pharmacokinetic data of pellitorine and sarmentine.

### 3.3. Pharmacokinetic Parameters of the Markers

**Figure 1:**
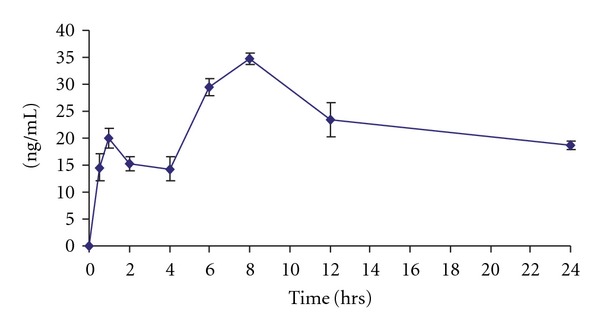
Pharmacokinetic profile of pellitorine after administering oral dose of 500 mg kg^−1^ of fruit ethanol extract of *P. sarmentosum* (each point is mean of six rats ± SD).

**Figure 2:**
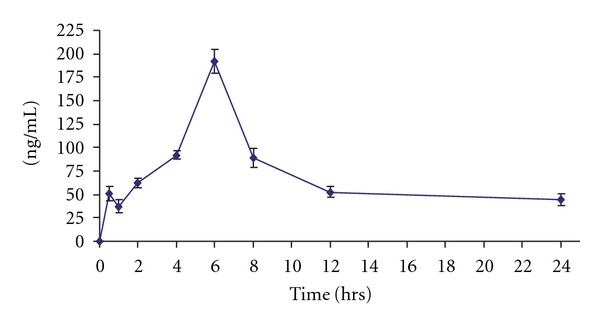
Pharmacokinetic profile of sarmentine after administering oral dose of 500 mg kg^−1^ of fruit ethanol extract of *P. sarmentosum* (each point is mean of six rats ± SD).

The results of pharmacokinetic parameters of pellitorine and sarmentine are presented in Tables 2 and 3, respectively. These results indicated that pellitorine exhibited *C*_max_ 34.77 ± 1.04 ng mL^−1^, *T*_max_ 8 h, MRT 26.00 ± 0.149 h and *t*_1/2_ 18.64 ± 1.65 h, whereas sarmentine showed *C*_max_ 191.50 ± 12.69 ng mL^−1^, *T*_max_ 6 h, MRT 11.12 ± 0.44 h and *t*_1/2_ 10.30 ± 1.98 h. The plasma concentration versus time profiles of pellitorine and sarmentine are presented in Figures 1 and 2, respectively. It is evident from these results that sarmentine stays in the body for lesser time as compared with pellitorine. Sarmentosine exhibited zero oral bioavailability because it was neither detected in plasma nor in tissues, feces and urine.

### 3.4. Tissue Distribution of the Marker Compounds

**Figure 3:**
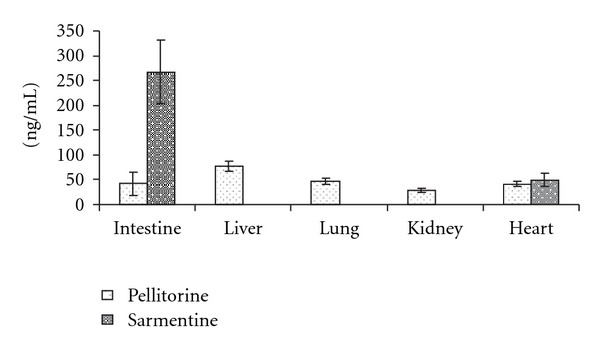
Concentration of pellitorine and sarmentine in different tissues of the rats (*n* = 3) at 6 h after administering oral dose (500 mg kg^−1^) of ethanol extract of fruit of *P. sarmentosum*.

The tissue distribution profiles of the markers in different tissues are presented in Figure 3. These results showed that pellitorine and sarmentine had different affinities toward different tissues. Pellitorine was found in intestinal wall, liver, lungs, kidney, and heart, whereas sarmentine was found in intestinal wall and heart.

### 3.5. Excretion of the Markers in Urine and Feces

The chromatograms of urine samples indicated that pellitorine and sarmentine were not excreted in urine as unchanged. It was expected that both the markers were metabolized to polar compounds to be excreted in urine. The same was noticed from chromatograms of urine samples, which indicated the increase in polarity of the samples that were collected after 5 h. Moreover, the polarity of urine samples was observed to becoming normal after 72 h.

The effect of the extract on urine output in 24 h presented in Table 4 indicated that there was not any significant difference in urine volume in both experimental group and control group (*P *< .05).

**Figure 4:**
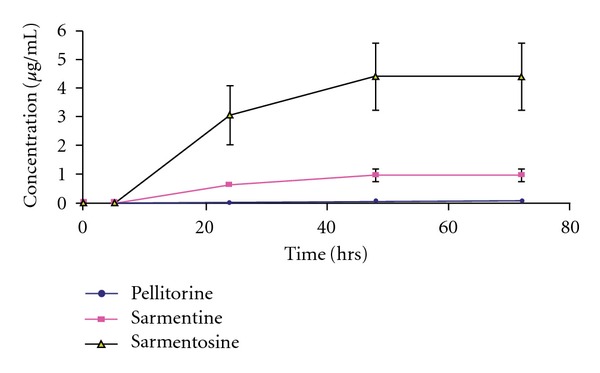
Excretion profiles of pellitorine, sarmentine and sarmentosine in feces after oral dose (500 mg kg^−1^) of ethanol extract of fruit of *P. sarmentosum* in rats.

The results of excretion of the markers in feces presented in (Table 4) indicated that cumulative excretion of pellitorine 0.0773 *μ*g in 0–72 h, which was 0.0007% of the oral dose. The oral bioavailability of pellitorine is considered good because fewer amounts are excreted in feces. The cumulative excretion of sarmentine was 0.976 *μ*g in 0–72 h, which was 0.0037% of the oral dose. This marker also exhibited good bioavailability but relatively lesser than pellitorine. The cumulative excretion of sarmentosine was 0.4377 *μ*g in 0–72 h, which was 0.94% of the dose. This marker exhibited zero oral bioavailability because it was excreted in feces as unchanged. The comparison of cumulative excretion profile of the markers in feces in 72 h is presented in Figure 4, which indicated that maximum excretion occurred 48 h after dosing.

## 4. Discussion

Keeping in view the versatile biological activities of extracts of the plant, pharmacokinetic studies based on three markers, pellitorine, sarmentine and sramentosine, were carried out in rats to delineate their absorption, distribution, metabolism and excretion after administering the extract orally. The oral route has certain merits and demerits, and oral drug absorption is affected by a number of factors; in gastric lumen drug may be metabolized by enzymes and microbial flora, inactivated by gastric contents and excreted in feces. The fraction of the drug which is absorbed may be metabolized in intestinal wall and in liver. Hence, the amount of drug which reaches in systemic circulation is lesser as compared with the administered dose. The action of the drug depends on the rate and extent at which drug reaches at the site(s) of action. Therefore, oral bioavailability data of the extract may be beneficial for its safe and effective use. Pharmacokinetic parameters help to understand the action of the body on the drug, which have numerous useful applications both in toxicology and biopharmaceutics. In present study, we have selected the oral route because the plant and its products are taken orally. The areas under curves of plasma concentration of pellitorine and sarmentine versus time have shown that drug exposure is long, which signifies the need of both the control of dose quantity and dosing interval.

**Figure 5:**
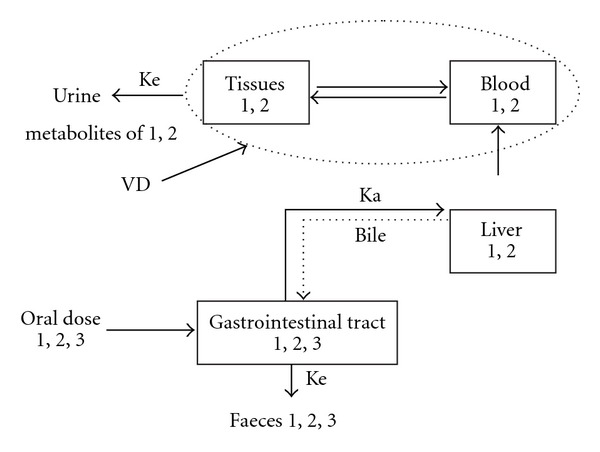
Pharmacokinetic model of pellitorine, sarmentine and sarmentosine after administering ethanol extract of fruit of *P. sarmentosum* in rats, *K*_a_ (absorption rate constant); *K*_e_ (constant elimination); VD (volume of distribution); 1 (pellitorine); 2 (sarmentine); 3 (sarmentosine).

On the basis of the results of this study, the proposed model for the pharmacokinetics of pellitorine, sarmentine and sarmentosine is given in (Figure 5). The drugs absorbed from gastrointestinal tract lead to liver, where these are biotransformed and delivered into blood stream to reach other organs. The appearance of pellitorine and sarmentine in various tissues indicates that these markers are either not metabolized or less metabolized in the liver. The absorbed drug is excreted through various routes but kidneys are the major organs involved in the excretion of most of drugs. In this study, pellitorine and sarmentine were not detected in urine, which indicated that either the markers were below the detection limit or in the form of metabolites. Based on chromatographic profiles, it was observed that both the markers were converted into polar metabolites to be excreted via urine. The polarity of the urine was observed to be increasing after 5 h of the dose administration. The polarity was found to be normal in samples that were collected after 72 h. It was found that these markers were excreted in urine in the form of metabolites. 

From the blood, drug distributes itself into various tissues based on physicochemical properties of the drug itself, effective tissue perfusion and behavior of cell membranes of the tissues. Selective tissue distribution of a drug is of a great value in targeting specific tissues and organs. The two absorbed markers have shown different affinities toward different tissues.

It is observed from the plasma concentration versus time profiles of the absorbed markers that pellitorine declines from 1 to 4 h followed by rise with maximum at 8 h while sarmentine declines from 30 min to 1 h followed by rise with maximum concentration at 6 h. The fluctuation in the plasma level time curve may be ascribed to a couple of pharmacokinetic phenomena such as hepatic-cycling, absorption from multiple-window and tissue distribution. The presence of outlier(s) may also be expected however, a consistent profile in all six animals excludes the probability of outlier(s) in the plasma level time curve for these markers. The fluctuation in plasma concentration versus time profile is found to be due to tissue distribution, which is apparent from tissue distribution profiles.

Sarmentosine is neither detected in plasma nor in tissues therefore, may it be assumed that either this marker is destroyed in gastrointestinal tract or excreted in the feces. It becomes evident from the analysis of feces that this marker is excreted unchanged without any absorption, and if absorbs, may be lesser in amount which is below the limit of detection of this method. This study suggests that this marker should be administered using other routes of administration or need to be modified to enhance its oral absorption.

It is evident from the study that the two markers of the extract, pellitorine and sarmentine, have good oral bioavailability and different tissue affinities, and are excreted in urine as metabolites. The other marker, sarmentosine, is excreted unchanged in feces and is not absorbed from the intestine.

## Figures and Tables

**Table 1:** Results of calibration, LOD, and LOQ of pellitorine, sarmentine and sarmentosine by HPLC with UV detection at 260 nm.

Standards	Linear regression equation	*R* ^2^	Linear range (ng mL^−1^)	LOD (ng mL^−1^)	LOQ (ng mL^−1^)
Pellitorine	*Y* = 0.2156*X*−0.0333	1.0000	10–500	3.00	10.00
Sarmentine	*Y* = 0.0985*X*−1.2335	0.9979	10–1500	3.00	10.00
Sarmentosine	*Y* = 0.0424*X*−1.4979	0.9999	80–12000	20.00	80.00

**Table 2:** Pharmacokinetic parameters of pellitorine in rats (*n* = 6) following an oral dose (500 mg kg^−1^) of ethanol extract of fruit of *P. sarmentosum*.

Rat	1	2	3	4	5	6	Mean ± SD
AUC_0-∞_ (ng h mL^−1^)	1154.352	989.1177	1013.913	979.7302	978.4666	1107.007	1037.098 ± 75.10543
AUMC_0-∞_	29 997.640	25 654.080	26 668.00	25 454.990	25 399.670	28 650.570	26 970.830 ± 1927.431
MRT (h)	25.987	25.937	26.302	25.982	25.959	25.881	26.008 ± 0.149
*T*_1/2_ (h)	21.397	17.617	17.290	19.045	17.087	19.436	18.645 ± 1.654
*C*_ max_ (ng mL^−1^)	33.880	33.420	36.310	34.710	34.870	35.460	34.775 ± 1.047
*T*_ max_ (h)	8.000	8.000	8.000	8.000	8.000	8.000	8.000 ± 0.000
*K*_ el_ (h^−1^)	0.033	0.039	0.040	0.036	0.046	0.037	0.038 ± 0.005
Cl	0.065	0.085	0.0751	0.086	0.087	0.071	0.078 ± 0.008
VD	1.993	2.158	1.877	2.355	2.135	1.972	2.082 ± 0.156

**Table 3:** Pharmacokinetic parameter of sarmentine in rats following an oral dose (500 mg kg^−1^) of ethanol extract of fruit of *P. sarmentosum*.

Rat	1	2	3	4	5	6	Mean ± SD
AUC_0-∞_ (ng h mL^−1^)	5078.443	5304.598	4674.769	4684.233	4749.764	4900.847	4898.776 ± 251.1527
AUMC_0-∞_	55 489.980	60 942.670	55 318.870	51 450.740	51 587.150	52 239.930	54 504.890 ± 3634.530
MRT (h)	10.927	11.489	11.834	10.984	10.861	10.659	11.126 ± 0.443
*T*_1/2_ (h)	10.380	11.873	13.183	7.668	9.714	9.023	10.307 ± 1.985
*C*_ max_ (ng mL^−1^)	196.547	194.318	166.476	194.929	194.54	202.669	191.580 ± 12.691
*T*_ max_ (h)	6.000	6.000	6.000	6.000	6.000	6.000	6.000 ± 0.000
*K*_ el_ (h^−1^)	0.089	0.078	0.070	0.121	0.095	0.103	0.093 ± 0.018
Cl	0.004	0.004	0.004	0.005	0.005	0.004	0.004 ± 0.000
VD	0.042	0.052	0.059	0.038	0.048	0.039	0.047 ± 0.008

**Table 4:** Cumulative excretion of pellitorine, sarmentine and sarmentosine in feces after oral dose of 500 mg kg^−1^ of ethanol extract of fruit of *P. sarmentosum*, and outcome of the extract on urine volume in experimental and control groups.

Excretion parameters	Rat 1	Rat 2	Rat 3	Mean	SD
Excretion of pellitorine					
Cumulative amount in *μ*g (0–72 h)	0.091	0.0564	0.0845	0.0773	0.0183
Percent of dose	0.001	0.001	0.001	0.001	0.000
Peak time (h)	48.000	48.000	48.000	48.000	0.000
Maximum excretion rate (*μ*g h^−1^)	0.002	0.002	0.002	0.002	0.000
Excretion of sarmentine					
Cumulative amount in *μ*g (0–72 h)	1.2176	0.866	0.844	0.976	0.2093
Percent of dose	0.0041	0.0036	0.0035	0.0037	0.0003
Peak time (h)	24.000	24.000	24.000	24.000	0.000
Maximum excretion rate (*μ*g h^−1^)	0.026	0.028	0.025	0.026	0.002
Excretion of sarmentosine					
Cumulative amount in *μ*g (0–72 h)	5.206	3.046	4.882	4.377	1.165
Percent of dose	1.117	0.653	1.047	0.939	0.249
Peak time (h)	24.000	24.000	24.000	24.000	0.000
Maximum excretion rate (*μ*g h^−1^)	0.155	0.078	0.148	0.127	0.043
Urine volume in experimental group					
Cumulative urinary volume in mL (0–24 h)	14.520	18.350	16.750	16.540	1.924
Maximum urine flow rate (mL h^−1^)	0.605	0.765	0.697	0.689	0.081

	Control 1	Control 2	Control 3	Mean	SD

Urine volume in control group					
Cumulative urinary volume in mL (0–24 h)	15.670	17.340	14.430	15.813	1.461
Maximum urine flow rate (mL h^−1^)	0.653	0.723	0.602	0.659	0.061

Each value represents the mean of three rats ± SD.
